# Blood Vessel Invasion as a Strong Independent Prognostic Indicator in Non-Small Cell Lung Cancer: A Systematic Review and Meta-Analysis

**DOI:** 10.1371/journal.pone.0028844

**Published:** 2011-12-14

**Authors:** Jun Wang, Jianpeng Chen, Xi Chen, Baocheng Wang, Kainan Li, Jingwang Bi

**Affiliations:** 1 Department of Oncology, General Hospital, Jinan Command of the People's Liberation Army, Jinan, China; 2 Department of Oncology, Provincial Hospital, Shandong University, Jinan, China; 3 Department of Internal Medicine, Huangsi Aesthetic Surgery Hospital, Beijing, China; Lerner Research Institute, Cleveland Clinic, United States of America

## Abstract

**Background and Objective:**

Blood vessel invasion plays a very important role in the progression and metastasis of cancer. However, blood vessel invasion as a prognostic factor for survival in non-small cell lung cancer (NSCLC) remains controversial. The aim of this study is to explore the relationship between blood vessel invasion and outcome in patients with NSCLC using meta-analysis.

**Methods:**

A meta-analysis of published studies was conducted to investigate the effects of blood vessel invasion on both relapse-free survival (RFS) and overall survival (OS) for patients with NSCLC. Hazard ratios (HRs) with 95% confidence intervals (95% CIs) were used to assess the strength of this association.

**Results:**

A total of 16,535 patients from 52 eligible studies were included in the systematic review and meta-analysis. In total, blood vessel invasion was detected in 29.8% (median; range from 6.2% to 77.0%) of patients with NSCLC. The univariate and multivariate estimates for RFS were 3.28 (95% CI: 2.14–5.05; *P*<0.0001) and 3.98 (95% CI: 2.24–7.06; *P*<0.0001), respectively. For the analyses of blood vessel invasion and OS, the pooled HR estimate was 2.22 (95% CI: 1.93–2.56; *P*<0.0001) by univariate analysis and 1.90 (95% CI: 1.65–2.19; *P*<0.0001) by multivariate analysis. Furthermore, in stage I NSCLC patients, the meta-risk for recurrence (HR = 6.93, 95% CI: 4.23–11.37, *P*<0.0001) and death (HR = 2.15, 95% CI: 1.68–2.75; *P*<0.0001) remained highly significant by multivariate analysis.

**Conclusions:**

This study shows that blood vessel invasion appears to be an independent negative prognosticator in surgically managed NSCLC. However, adequately designed large prospective studies and investigations are warranted to confirm the present findings.

## Introduction

Non-small cell lung cancer (NSCLC) constitutes approximately 80% of all cases of primary lung cancers and is one of the most common tumors worldwide [1]. Although surgical resection is the current standard of treatment for early-stage patients, only 15% of patients diagnosed with NSCLC survive for 5 years [2]. Prognostic factors may identify subsets of patients with a worse prognosis and facilitate the selection of a more aggressive treatment strategy. The tumor-node-metastasis (TNM) staging system is the best prognostic index for operable NSCLC [2]. However, each patient's prognosis varies significantly within each TNM stage, which makes it difficult to predict accurately the outcome for each patient, especially for patients with early-stage lung cancer.

The degree of histological differentiation, extent of operation, visceral pleural invasion, and many biological factors involving in cancer development and progression are useful for predicting survival and aiding the management of patients with NSCLC [3]–[5]. Our previous meta-analysis concluded that the methylation of *RASSF1A* within its promoter region could serve as an independent prognostic marker for NSCLC [6]. In the latest edition (7th) of the TNM classification, tumor size is evaluated in detail, with visceral pleural invasion clearly defined; T1 tumors are still classified as T2 if the visceral pleura elastic layer is invaded [1]. In testicular germ cell tumors, cancer cell invasion of blood vessels qualifies as a local spread of tumors. Testicular tumors localized to the testis and epididymis are classified as T1 when blood vessel invasion (BVI) is absent, whereas they are upgraded to T2 if vascular invasion is present [7].

In 1992, Macchiarini *et al.* first demonstrated that NSCLC patients harboring BVI have a significant tendency to relapse in the first 5-year period after surgery and would be suitable for adjuvant chemotherapy [8]. In the last decade, BVI has also been reported to be a strong predictor of recurrence [9]–[19] or death for patients with NSCLC in many studies [8]–[10], [12], [16], [17], [20]–[47], but has not been confirmed in other studies [48]–[55]. Based on the discordant results obtained by a large number of studies on lung cancer, we performed a literature-based systematic review to better quantify the prognostic effects of BVI on both relapse-free survival (RFS) and overall survival (OS) in patients with NSCLC.

## Materials and Methods

### Publication selection, data extraction and methodological assessment

Studies were identified via a search of the electronic databases PubMed (National Library of Medicine, Bethesda, USA) and EMBASE (Elsevier, Amsterdam, the Netherlands) between 1980 and 2011 using the following key words: non-small cell lung cancer, NSCLC, vessel invasion, vascular invasion, relapse, recurrence, prognostic and prognosis (last search was updated on July 10, 2011). To be eligible for inclusion, studies had to meet the following criteria: (i) blood vessel invasion (not lymphovascular invasion) was measured in surgically resected primary tumor samples, and (ii) the relationship between BVI and survival was investigated, and the results were published as a full paper.

Abstracts, reviews and case reports were excluded because of insufficient data for meta-analysis. Non-English language papers were not included in the review. To avoid duplication of patient data, we carefully noted the author names and the research centers involved for all authors. If more than one publication reported the same population data, only the most recently reported data or complete data were used. We also performed a manual search from the references of relevant publications, including original articles and reviews, to identify additional studies. Three authors (J. Wang, J. Chen and X. Chen) did the search and identification independently using a standardized approach, and the selection of a study was reached by consensus. Information retrieved from the reports included author names, year of publication, patient resources, study size, methods, histology, and disease stage. Methodological assessment was also independently performed by three investigators (J. Wang, J. Chen and X. Chen). Disagreements were adjudicated by a third investigator (B. Wang) after referring to the original articles. Scoring for each study was conducted according to the European Lung Cancer Working Party scale by Steels *et al* [3]. Studies included in the systematic review were denoted ‘eligible’, and those providing sufficient data for the meta-analysis are denoted ‘evaluable’.

### Statistical methods

We performed separate meta-analyses using an adjusted or unadjusted hazard ratio (HR) for RFS and OS. Usually, HRs and 95% confidence intervals (CIs) were directly obtained from published literatures using univariate [8]–[11], [13]–[15], [18], [19] or multivariate survival analyses. In some studies, BVI was determined to be an independent prognostic indicator using multivariate analysis; HRs and 95% CIs were generally reported [9], [10], [16], [18], [20], [21], [23], [26]–[29], [31], [33]–[36], [38], [40]–[45], [47]–[49], [52], [56]–[59], [60]. Some studies reported the HR but did provide sufficient information on survival by BVI status; we thus estimated the HR and CIs according to the methods of Parmar *et al* [61]. As shown in Table S1, the total number of events [53], the log-rank statistic or its *P* value [9]–[12], [14], [17], [18], [20]–[22], [25], [27], [28], [34], [35], [39]–[42], [44], [46], [47], [50], [51], [54], [57], [58], or data from Kaplan-Meier survival curves [16]–[19], [59] were used to allow an approximate calculation of the HR. Heterogeneity was tested with the χ^2^-based *Q* test [62]. When the effects were assumed to be homogenous, the fixed-effect model was used; otherwise, the random-effect model based on Mantel–Haenszel method was applied [36] A funnel plot and Egger's linear regression test were used to investigate any possible publication bias [63]. The correlation between the score measurements was determined using the Spearman rank correlation coefficient. The score measurements involving the value of a discrete variable were calculated using the nonparametric Mann-Whitney U test. For all analyses, a two-sided *P* value of <0.05 was considered statistically significant. All analyses were performed using STATA version 11.0 software (Stata Corporation, College Station, TX, USA).

## Results

### Study selection and characteristics

In total, our electronic data search retrieved 206 references. Four studies were excluded because an identical patient cohort occurred within another selected cohort [64]–[67]. Eight studies were not included in the overall meta-analysis because they investigated lymphovascular invasion and outcome in NSCLC patients. The other excluded records include 19 reviews, 3 other diseases, 3 case reports, 13 non-English articles and 103 studies without available survival information (Table S2). Macchiarini *et al.* analyzed the correlation between BVI and outcome in 28 patients receiving induction therapy [68]. This study was also excluded from the systematic review because it did not meet the inclusion criteria. Finally, there were 52 eligible studies investigating the prognostic value of BVI in patients with NSCLC published from 1993 to 2011. The PRISMA Checklist and Flow Diagram for the studies is shown in Checklist S1 and Figure S1.

The individual characteristics of the 52 eligible studies are reported in Table S3. The total number of patients was 16,535 (range, 35–2295; median, 171). In all, according to the positivity for BVI as defined by the authors, 29.8% of tumors harbored BVI by cancer cells (median; range from 6.2% to 77.0%). A total of 47 studies dealt with all types of NSCLC, four with adenocarcinoma alone [14], [27], [38], [41] and one with squamous cell carcinoma alone [39]. A total of 23 studies dealt with only stage I patients. Twenty-eight studies investigated blood vascular invasion by using hematoxylin and eosin (H&E) stain alone. Three studies investigated blood vascular invasion by staining with H&E and for CD34 with or without CD31 immunochemistry [12], [50], [53]. Twenty studies evaluated the status of BVI in tumors in combination with elastic staining.

Of the 52 publications eligible for systematic review, four were not evaluable by meta-analysis owing to the lack of survival data even after writing to the authors for complementary information [30], [32], [53], [55]. Twelve of the these eligible studies identified BVI as a poor prognostic factor for RFS [8], [10]–[14], [16], [18], [19], [21], [60], [69], and three identified BVI as not significant for RFS [35], [53], [54] by univariate analysis. However, three studies were not included in final meta-analysis due to insufficient survival information [17], [53] or overlap between cohorts [10], [11]. Eight studies reported the significant prognostic role of BVI for RFS by multivariate analysis [8], [9]–[11], [13]–[15], [19], [60], and two identified BVI as not significant for RFS [18], [54].

In univariate analysis, 33 studies identified blood vessels as a significant prognostic factor for OS [8], [9], [12], [20]–[25], [27]–[34], [43]–[47], [58], [16], [17], [35], [37]–[42], [59], and seven identified it as not significant for survival [18], [50], [52]–[55], [70]. In multivariate analyses, there were 27 studies that identified BVI as a significant prognostic factor for OS [8]–[10], [16], [20], [23], [26], [27], [31]–[47], [58], [59] and 10 that identified blood vessel as a non-significant prognostic factor [18], [21], [28], [30], [48], [49], [52], [53], [56], [70]. Evaluability was not associated with positivity in the systematic review. The rate of significant results for OS was 62.7% for evaluable trials (33/52) compared with 60.0% (2/4) for non-evaluable trials (*P* = 0.626) irrespective of whether these studies used univariate or multivariate analyses.

### Quality assessment of study

As shown in Table 1, the global quality assessment score, expressed as a percentage, ranged from 46.5% to 63.6%. There was no significant association between the global score and the number of patients in all eligible studies (*P* = 0.32). As for the global score, no significant difference was found between the evaluable and the non-evaluable trials (*P* = 0.18). Similarly, no statistically significant difference was shown between the significant trials and the non-significant trials in univariate (*P* = 0.86) or multivariate analysis for OS (*P* = 0.52) (Table 1).

**Table 1:** Quality scores analysis of the 52 eligible studies by the European Lung Cancer Working Party score according to studies characteristics.

Studies	Design (/10)	Laboratory method (/10)	Generalizability (/10)	Results analysis	Global score (%)
All (52)	4.0	5.6	6.4	6.0	55.0
Patients number					
Spearman *r*	0.17	0.14	0.10	0.25	0.14
*P*	0.22	0.32	0.47	0.08	0.32
Evaluable for meta-analysis					
Yes (48)	4.0	4.2	6.4	7.5	58.9
No (4)	4.0	5.6	6.4	5.0	52.5
*P*	0.38	0.15	0.13	0 .36	0.18
Significant results for OS in univariate analysis					
Yes (33)	4.0	4.9	5.6	7.5	59.5
No (7)	6.0	4.2	6.4	7.5	60.2
*P*	0.78	085	0.98	0.81	0.86
Significant results for OS in multivariate analysis					
Yes (27)	4.0	5.6	6.4	7.0	55.3
No (10)	4.0	5.6	6.4	6.3	53.6
*P*	0.36	0.91	0.91	0.63	0.52

Scores distributions are summarized by median values.

OS = overall survival.

### Meta-analysis of the impact of BVI on survival for all patients

**Figure 1:**
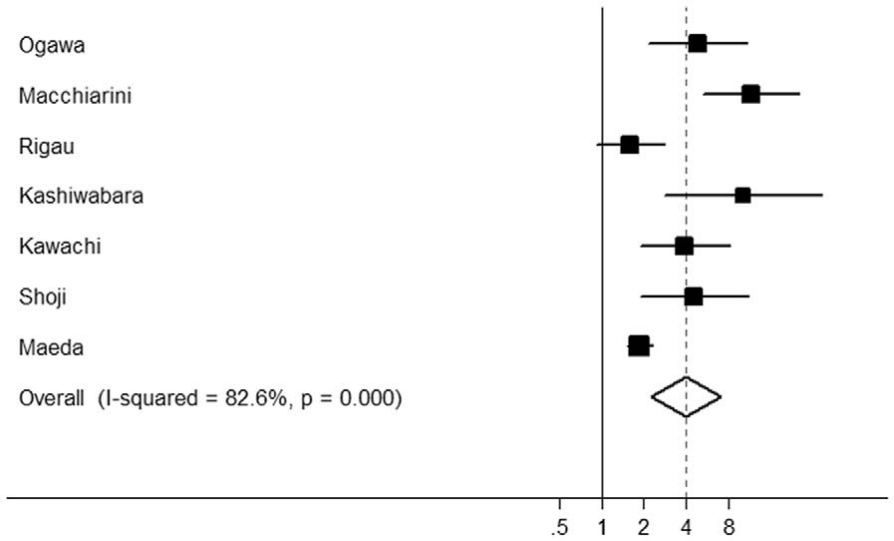
Forest plot showing the combined relative hazard ratio for relapse-free survival in all patient populations by multivariate analysis.

The results of meta-analysis of BVI and prognosis in NSCLC are shown in Table 2. BVI was significantly correlated with poor RFS according to univariate analysis, with a combined HR of 3.28 (95% CI: 2.14–5.05; *P*<0.0001) (11 studies, 4,498 patients). Significant heterogeneity was detected among these studies (*P*<0.001, *Q* = 90.00). The data of multivariate analyses for RFS were available in six studies including 3,088 patients. We obtained a combined HR value of 3.98 (95% CI: 2.24–7.06; *P*<0.0001) with significant heterogeneity (*P*<0.001, *Q* = 34.44) (Fig. 1).

**Table 2:** Results of meta-analysis of blood vessel invasion and prognosis in NSCLC.

Groups	Estimate of relative hazard			Homogeneity test	
	HR	95% CI	*p*	*Q* (df)	*p*
All studies					
Unadjusted OS (31 studies, n = 8,528)	2.22	1.93–2.56	<0.0001	79.63 (33)	<0.001
Adjusted OS (28 studies, n = 9,873)	1.90	1.65–2.19	<0.0001	84.40 (28)	<0.001
Unadjusted RFS (11 studies, n = 4,498 )	3.28	2.14–5.05	<0.0001	90.00 (10)	<0.001
Adjusted RFS (7 studies, n = 3,088)	3.98	2.24–7.06	<0.0001	34.44 (6)	<0.001
Stage I studies					
Unadjusted OS (14 studies, n = 2,908 )	2.94	2.28–3.80	<0.0001	22.83 (13)	0.044
Adjusted OS (13 studies, n = 3,974)	2.15	1.68–2.75	<0.0001	26.70 (12)	0.009
Unadjusted RFS (5 studies, n = 647)	5.89	3.98–8.71	<0.0001	3.76 (4)	0.440
Adjusted RFS (4 studies, n = 576)	6.93	4.23–11.37	<0.0001	3.61 (3)	0.306

NSCLC = non-small-cell lung cancer; HR = hazard ratio; CI = confidence interval; OS = overall survival; RFS = relapse-free survival.

**Figure 2:**
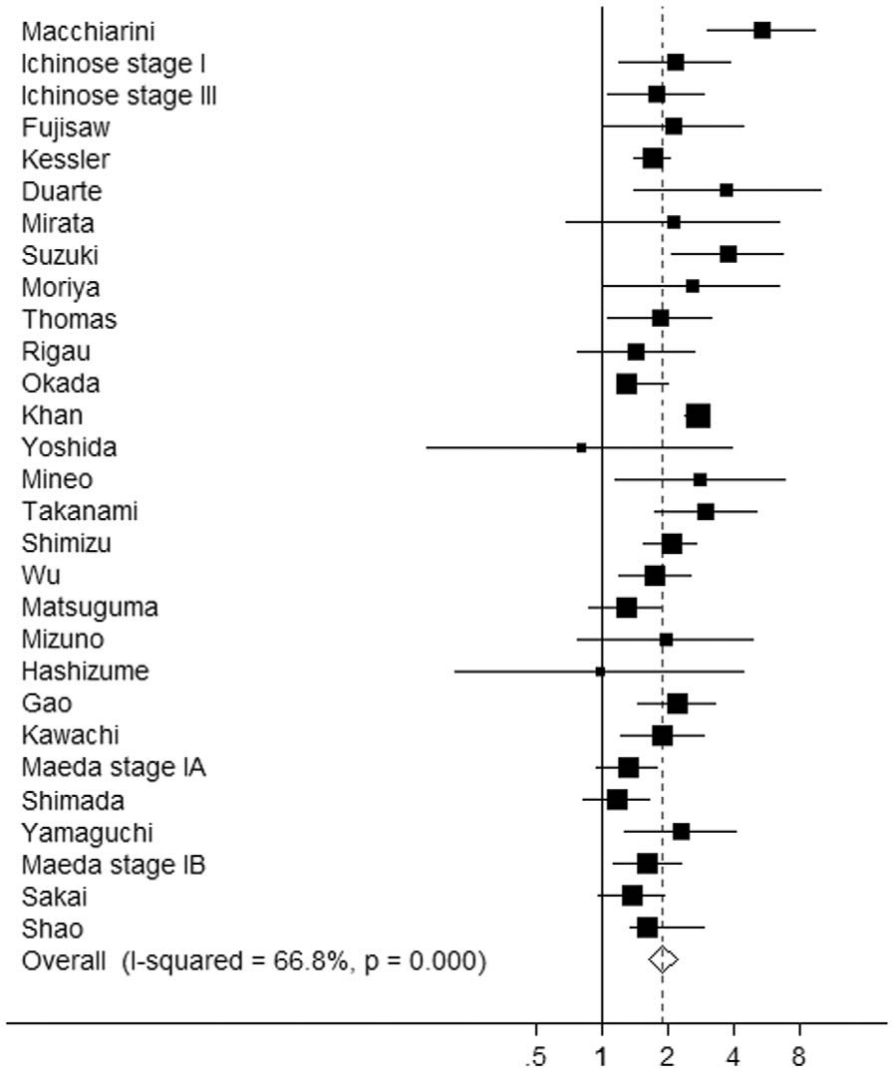
Forest plot showing the combined relative hazard ratio for overall survival in all patient populations by multivariate analysis.

Thirty-one studies (comprising 8,528 cases) used for univariate analysis produced a pooled estimate of risk of 2.22 (95% CI: 1.93–2.56, *P*<0.0001). There was evidence of significant inter-study heterogeneity (*P*<0.001, *Q* = 79.63). Twenty-eight studies (comprising 9,873 cases) were used for the meta-analysis of BVI on OS by multivariate analysis (Fig. 2). The overall risk of death was 1.90 (95% CI: 1.65–2.19; *P*<0.0001) with a range of estimates from 0.80 to 3.79. Significant heterogeneity among studies was present (*P*<0.001, *Q* = 84.40).

### Meta-analysis of the impact of BVI on survival for stage I patients

**Figure 3:**
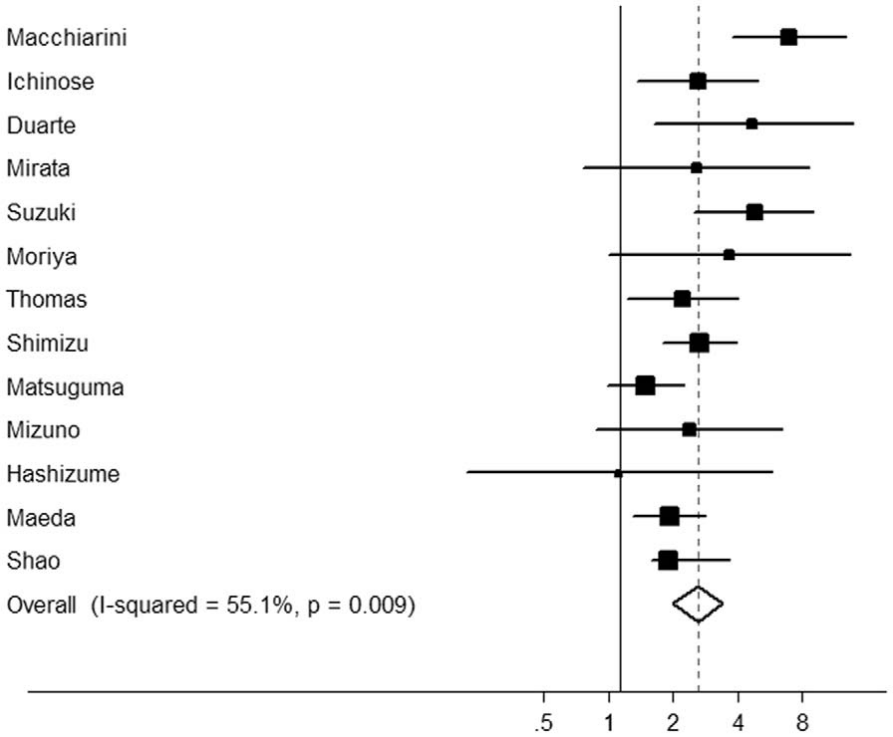
Forest plot showing the combined relative hazard ratio for overall survival of stage I patients by multivariate analysis.

We also analyzed the association between the BVI and survival in early-stage cancer patients. As shown in Table 2, the summary HR estimates for stage I patients by univariate and multivariate analysis were 2.94 (95% CI: 2.28–3.80; *P*<0.0001) and 2.15 (95% CI: 1.68–2.75; *P*<0.0001) , respectively. BVI demonstrated a significant effect on RFS for stage I NSCLC according to univariate analysis (HR = 5.89, 95% CI: 3.98–8.71, *P*<0.0001) and multivariate analysis (HR = 6.93, 95% CI: 4.23–11.37, *P*<0.0001) (Fig. 3). These results suggest that NSCLC patients with BVI have a poor prognosis, irrespective of the tumor stage.

### Test of heterogeneity and subgroup analyses

There was large heterogeneity in this meta-analysis. Firstly, we performed the subgroup analyses stratified by ethnicity. BVI status did show a significant effect on RFS according to multivariate analysis in Asians (HR = 3.87, 95% CI: 2.07–7.22, *P*<0.001; heterogeneity test, *P* = 0.006) and in Caucasians (HR = 4.25, 95% CI: 0.61–29.5, *P*<0.001; heterogeneity test, *P* = 0.28). There was still evidence of statistical heterogeneity in Asians that was largely due to the contribution of the report by Maeda *et al.* which included the largest patient population. Furthermore, the heterogeneity disappeared when excluding this study and the value of pooled HR was not significantly altered (HR = 4.82, 95% CI: 3.12–7.46, *P*<0.001; heterogeneity test, *P* = 0.65). Subgroup analyses by methods of BVI evaluation showed that the pooled HRs for RFS by multivariate analysis were 4.91 (95% CI: 1.82–13.24, *P*<0.001; heterogeneity test, *P* = 0.06) in studies evaluating BVI with H&E method and 3.16 (95% CI: 1.51–6.63, *P*<0.001; heterogeneity test, *P* = 0.02) in those evaluating BVI by a combination of H&E method and immunochemistry or elastic staining.

In the subgroup analysis by ethnicity, statistically significantly increased risks for death were found among Asians (HR = 1.68, 95% CI: 1.53–1.85, *P*<0.001; heterogeneity test, *P* = 0.06) and among Caucasians (HR = 1.74, 95% CI: 1.46–2.09, *P*<0.001; heterogeneity test, *P* = 0.45). In addition, BVI was significantly correlated with poor OS according to multivariate analysis in studies evaluating BVI with H&E method (HR = 2.04, 95% CI: 1.68–2.47, *P*<0.001; heterogeneity test, *P* = 0.03) and in those evaluating BVI by a combination of H&E method and immunochemistry or elastic staining (HR = 1.60, 95% CI: 1.39–1.84, *P*<0.001; heterogeneity test, *P* = 0.28). These results showed that the heterogeneities were effectively decreased or removed in the subgroups by ethnicity and methods for BVI evaluation.

Publication bias statistics were determined using the methods of Egger *et al* [63]. No publication bias was found for the studies used for univariate analysis (*P* = 0.596) or for multivariate analysis (*P* = 0.24).

## Discussion

Microscopic metastasis begins with the local invasion by tumor cells into host stroma within or surrounding the primary tumor. When tumor cells penetrate a blood vessel or a peripheral lymphatic, they can detach, disseminate and arrest in the microvasculature through the circulation [70]. In fact, the presence of vascular invasion by neoplastic cells indicates that the cancers are in a metastatic phase. BVI was found to correlate with disease progress in many types of cancers [71]–[73]. Our meta-analysis provides strong evidence that pathologic BVI is a prognostic factor for survival in patients with NSCLC by univariate analysis. More importantly, this effect remained significant according to multivariate analysis, showing that BVI is an independent prognostic factor for poor survival regardless of tumor size or lymph node status. In addition, vascular invasion is a strong predictor of survival in early stage tumors when adjusted for other prognostic factors.

Cancer metastasis and progression is a complex and multistep process. BVI appears to be a fundamental step in cancer metastasis and spread, leading to unfavorable prognosis for patients with NSCLC. In general, BVI was defined as tumor cell embolization in the vascular lumen on routine H&E and elastic lamina stain. Ichinose *et al.* reported that venous invasion correlated with poor prognosis among patients with completely resected NSCLC but that arterial invasion did not [47]. However, in most of the studies, arterial and venous invasion has not been separately studied pathologically due to the inability in some cases to discriminate between arterial and venous invasion. In addition, most of the studies included in this meta-analysis investigated the correlation between intratumoral BVI and prognosis, but some studies demonstrated the prognostic role of extratumoral vascular invasion. Shimada *et al.* reported that both intratumoral and extratumoral vascular invasion were significant prognostic factors, but only the extratumoral vascular invasion group was associated with advanced pathologic staging, lymph node metastasis, and lymphatic permeation [21]. Our meta-analysis focused on the effect of tumor vascular invasion on the survival of NSCLC patients irrespective of whether these studies detected intratumoral or extratumoral vascular invasion or whether they determined arterial and venous invasion. Although almost all intratumoral blood vessels are occluded by the surrounding tumor cells and stromal cells, intratumoral and extratumoral blood vessels are thought to be functional blood vessels. In fact, Shimada *et al.*, found that all extratumoral vascular invasion cases also had intratumoral vascular invasion [21].

Our meta-analysis had some limitations. First, the level of evidence obtained by retrospective studies is lower than that provided by randomized controlled trials. Second, data from published trials rather than individual patient data were used in the systematic review. Third, in most of meta-analyses, there was evidence of significant heterogeneity although the random-effect model based on Mantel–Haenszel method rather than the fixed-effect model was applied. Studies may have differed with regard to the baseline characteristics of the patients included, the adjuvant treatment they might have received, the duration of follow-up, and adjustments for other cofactors. For example, some studies included a small number of stage IV or IIIB patients, which accounted for the heterogeneity. These studies were finally maintained in the meta-analyses because the overall designs of studies were similar to those used in the other studies. Many variations to the method of BVI assessment exist, although in most studies BVI was defined as the presence of neoplastic structures inside the lumen of a vessel. Some studies detected BVI by staining with hematoxylin and eosin alone or in combination with elastic-van Gieson stain or by staining with Victoria blue hematoxylin and eosin, which can lead to significant heterogeneity. Subgroup analyses indicated that methods for BVI evaluation contributed largely to heterogeneity. Thus, standardization of evaluating vascular invasion and quality control is needed. In addition, an important issue that needs to be taken into account is the type of adjuvant therapy that each patient received because chemotherapy and/or therapies that target the epidermal growth factor receptor can change the outcome for NSCLC patients. However, the majority of published studies lacked detailed information regarding patient treatment. In this study, we used a methodology assessment on the treatment of lung cancer reported by Steels *et al* [3]. However, this approach does not fully protect from potential bias because we could not take all the studies into account. Therefore, our results need to be substantiated by further prospective studies.

Publication and reporting bias also has to be considered. We did not look for unpublished papers, reviews or abstracts because the required data were usually available only in full publications. Another potential source of bias is related to the method used to extrapolate the HR. If the HR was not reported by author, it was calculated from the data included in the article or extrapolated from the survival curves, which involves making assumptions. In addition, each study adjusted for different covariates and only the studies that found significant results in univariate analysis performed multivariate analysis; thus, pooling the results may have produced bias. Nevertheless, no publication bias was detected using Egger's test, suggesting that the statistics obtained approximate the actual results.

In conclusion, the relative risk of recurrence and death for an individual patient whose tumor showed BVI by neoplastic cells was nearly 4 and 2 times higher, respectively, than that of a patient whose tumor did not show BVI by neoplastic cells. We have demonstrated the prognostic power of a single independent pathologic marker for NSCLC. We suggest that NSCLC patients with BVI receive adjunct systematic chemotherapy and that BVI should be incorporated in the new edition of the TNM classification. However, large, properly designed studies that employ standard methodology to assess BVI are needed to demonstrate whether BVI can provide prognostic information in addition to the currently used TNM staging system. Moreover, according to the recent report by Kato *et al.*, the significance of a combination of microvessel count and BVI could be more important than that of either BVI or microvessel count alone [74].

## Supporting Information

Figure S1 The flow of the included studies.
(DOC)

Checklist S1 PRISMA checklist.
(DOC)

Table S1 Data source for the estimating of HR form included studies evaluating blood vessel invasion and prognosis.
(DOC)

Table S2 Characteristics of literatures excluded in this systematic review.
(DOC)

Table S3 Main characteristics and results of eligible studies evaluating blood vessel invasion and RFS or OS in patients with NSCLC.
(DOC)
