# Supplementary material for: Blood Vessel Invasion as a Strong Independent Prognostic Indicator in Non-Small Cell Lung Cancer: A Systematic Review and Meta-Analysis
Source: PLoS One. 2011 Dec 14;6(12):e28844. doi: 10.1371/journal.pone.0028844 (PMC3237541; doi:10.1371/journal.pone.0028844)
Supplement: Figure S1 — The flow of the included studies. (DOC) [file pone.0028844.s001.doc]

**PRISMA Flow Diagram**

**Screening**

**Included**

**Eligibility**

**Identification**

Records identified through database searching
(n = 206)

Additional records identified through other sources
(n = 0)

Records after duplicates removed
(n = 4)

Records screened
(n = 202)

Records excluded
(n =149; 19 reviews, 3 other diseases, 3 case reports, 13 non-English articles, 8 studies investigating lymphatic invasion and 103 studies without survival information)

Full-text articles assessed for eligibility
(n = 53)

Full-text article was excluded because patients received induction therapy
(n = 1)

Studies included in qualitative synthesis
(n = 52)

Studies included in univariate meta-analysis for RFS
(n = 11)

Studies included in multivariate meta-analysis for RFS
(n = 7)

Studies included in univariate meta-analysis for OS
(n = 31)

Studies included in multivariate meta-analysis for OS
(n = 28)
