# Supplementary material for: Blood Vessel Invasion as a Strong Independent Prognostic Indicator in Non-Small Cell Lung Cancer: A Systematic Review and Meta-Analysis
Source: PLoS One. 2011 Dec 14;6(12):e28844. doi: 10.1371/journal.pone.0028844 (PMC3237541; doi:10.1371/journal.pone.0028844)
Supplement: Table S1 — Data source for the estimating of HR form included studies evaluating blood vessel invasion and prognosis. (DOC) [file pone.0028844.s003.doc]

**Table S1.** Data source for the estimating of HR form included studies evaluating blood vessel invasion and prognosis

| First author | Year | RFS | | OS |  |
| --- | --- | --- | --- | --- | --- |
|  |  | Univariate | Multivariate analysis | Univariate | Multivariate analysis |
| Maeda *et al*. (60) | 2011 | HR, 95%CI | HR, 95%CI | N/A | N/A |
| Sakai *et al*. (56) | 2011 | N/A | N/A | N/A | HR, 95%CI |
| Shao *et al*. (20) | 2011 | N/A | N/A | *P*, event number | HR, 95%CI |
| Maeda *et al*. (11) | 2010 | *P*, event number | HR, 95%CI | N/A | HR, 95%CI |
| Shimada *et al*. (21) | 2010 | *P*, event number | N/A | *P*, event number | HR, 95%CI |
| Maeda *et al*. (11) | 2010 | *P*, event number | HR, 95%CI | N/A | N/A |
| Naito *et al*. (22) | 2010 | N/A | N/A | *P*, event number | N/A |
| Yamaguchi *et al*. (23) | 2010 | N/A | N/A | N/A | HR, 95%CI |
| Maeda *et al*. (9) | 2010 | N/A | N/A | *P*, event number | HR, 95%CI |
| Ryuge *et al*. (24) | 2010 | N/A | N/A | HR, 95%CI | N/A |
| Bodendorf *et al*. (25) | 2009 | N/A | N/A | *P*, event number | N/A |
| Kawachi *et al*. (26) | 2009 | N/A | N/A | N/A | HR, 95%CI |
| Turhan *et al*. (12) | 2009 | *P*, event number | N/A | *P*, event number | N/A |
| Shoji *et al*. (13) | 2009 | HR, 95%CI | HR, 95%CI | N/A | N/A |
| Mizuno *et al*. (27) | 2008 | N/A | N/A | *P*, event number | HR, 95%CI |
| Kashiwabara *et al*. (14) | 2008 | *P*, event number | HR, 95%CI | N/A | N/A |
| Hashizume *et al*. (28) | 2008 | N/A | N/A | *P*, event number | HR, 95%CI |
| Kawachi *et al*. (15) | 2008 | N/A | HR, 95%CI | N/A | N/A |
| Gao *et al*. (59) | 2008 | N/A | N/A | *P*, survival curves | HR, 95%CI |
| Rao *et al*. (29) | 2007 | N/A | N/A | *P*, event number | N/A |
| Takanami *et al*. (31) | 2005 | N/A | N/A | HR, 95%CI | HR, 95%CI |
| Shimizu *et al*. (58) | 2005 | N/A | N/A | *P*, event number | HR, 95%CI |
| Wu *et al*. (33) | 2005 | N/A | N/A | HR, 95%CI | HR, 95%CI |
| Yoshida *et al*. (49) | 2004 | N/A | N/A | N/A | HR, 95%CI |
| Mineo *et al*. (34) | 2004 | N/A | N/A | *P*, event number | HR, 95%CI |
| Yamamoto *et al*. (35) | 2004 | *P*, event number | N/A | *P*, event number | HR, 95%CI |
| Okada *et al*. (16) | 2003 | *P*, survival curves | N/A | *P*, survival curves | HR, 95%CI |
| Okada *et al*. (36) | 2003 | N/A | N/A | N/A | HR, 95%CI |
| Gabor *et al*. (17) | 2003 | *P*, event number | N/A | *P*, survival curves | N/A |
| Khan *et al*. (37) | 2003 | N/A | N/A | HR | HR, 95%CI |
| Rigau *et al*. (18) | 2002 | *P*, event number | HR, 95%CI | *P*, survival curves | HR, 95%CI |
| Moriya *et al*. (38) | 2001 | N/A | N/A | HR, 95%CI | HR, 95%CI |
| Tamura *et al*. (39) | 2001 | N/A | N/A | *P*, event number | HR |
| Thomas *et al*. (40) | 2001 | N/A | N/A | *P*, event number | HR, 95%CI |
| Yokose *et al*. (41) | 2000 | N/A | N/A | *P*, event number | HR, 95%CI |
| Cagini *et al*. (50) | 2000 | N/A | N/A | *P*, event number | N/A |
| Suzuki *et al*. (42) | 1999 | N/A | N/A | *P*, event number | HR, 95%CI |
| Fu et al. (51) | 1999 | N/A | N/A | *P*, event number | HR |
| Hirata et al (52) | 1998 | N/A | N/A | N/A | HR, 95%CI |
| Lucchi et al (53) | 1997 | event number | N/A | N/A | N/A |
| Kessler *et al*. (43) | 1996 | N/A | N/A | N/A | HR, 95%CI |
| Brechot *et al*. (54) | 1996 | *P*, event number | N/A | *P*, event number | N/A |
| Duarte *et al*. (44) | 1997 | N/A | N/A | *P*, event number | HR, 95%CI |
| Fujisaw *et al*. (45) | 1995 | N/A | N/A | HR, 95%CI | HR, 95%CI |
| Harpole *et al*. (65) | 1995 | N/A | N/A | *P*, event number | HR |
| Ichinose *et al*. (47) | 1994 | N/A | N/A | *P*, event number | HR, 95%CI |
| Ogawa *et al*. *et al*. (19) | 1993 | *P*, survival curves | HR, 95%CI | N/A | N/A |
| Macchiarini *et al*. (8) | 1993 | *P*, survival curves | HR, 95%CI | N/A | HR, 95%CI |

HR = hazard ratio; N/A = no available or no applicable; RFS = relapse-free survival; OS = overall survival.
