# Supplementary material for: Blood Vessel Invasion as a Strong Independent Prognostic Indicator in Non-Small Cell Lung Cancer: A Systematic Review and Meta-Analysis
Source: PLoS One. 2011 Dec 14;6(12):e28844. doi: 10.1371/journal.pone.0028844 (PMC3237541; doi:10.1371/journal.pone.0028844)
Supplement: Table S2 — Characteristics of literatures excluded in this systematic review. (DOC) [file pone.0028844.s004.doc]

**Table S2. Characteristics of literatures excluded in this systematic review**

| References | Year | Country | BVI evaluation | Exclusion Reasons |
| --- | --- | --- | --- | --- |
|
| [1] | 2011 | Japan | No | AC; BVI was not detected |
| [2] | 2010 | Spain | No | NSCLC; BVI was not detected |
| [3] | 2011 | Japan | No | NSCLC; BVI was not detected |
| [4] | 2011 | Korea | No | NSCLC; BVI was not detected |
| [5] | 2010 | USA | No | Review |
| [6] | 2011 | Japan | Yes | NSCLC; blood vessel or lymphatic invasion and survival |
| [7] | 2010 | Japan | Yes | NSCLC; survival analysis is not available |
| [8] | 2010 | Italy | No | NSCLC; BVI was not detected |
| [9] | 2010 | China | No | Esophageal carcinoma |
| [10] | 2010 | Japan | Yes | NSCLC, survival analysis is not available |
| [11] | 2010 | UK | No | NSCLC; BVI was not detected |
| [12] | 2011 | Japan | No | NSCLC; BVI was not detected |
| [13] | 2010 | China | Yes | NSCLC; survival analysis is not available |
| [14] | 2010 | Turkey | Yes | NSCLC; survival analysis is not available |
| [15] | 2010 | China | No | NSCLC; BVI was not detected |
| [16] | 2010 | USA | No | NSCLC; BVI was not detected |
| [17] | 2010 | India | No | Breast cancer |
| [18] | 2010 | Japan | No | NSCLC; BVI was not detected |
| [19] | 2009 | Japan | No | NSCLC; BVI was not detected |
| [20] | 2009 | Japan | No | NSCLC; BVI was not detected |
| [21] | 2010 | Japan | No | NSCLC; BVI was not detected |
| [22] | 2009 | France | No | Article in French |
| [23] | 2010 | Japan | No | NSCLC; BVI was not detected |
| [24] | 2009 | Turkey | No | Case report, NSCLC, BVI was not detected |
| [25] | 2009 | Japan | No | NSCLC; BVI was not detected |
| [26] | 2008 | Korea | No | NSCLC; BVI was not detected |
| [27] | 2009 | China | No | NSCLC; BVI was not detected |
| [28] | 2010 | Japan | No | NSCLC; BVI was not detected |
| [29] | 2009 | Japan | Yes | NSCLC; survival analysis is not available |
| [30] | 2009 | USA | No | Review |
| [31] | 2009 | The Netherlands | No | NSCLC; BVI was not detected |
| [32] | 2009 | China | Yes | NSCLC; Lymphovascular invasion and survival |
| [33] | 2009 | China | No | NSCLC; BVI was not detected |
| [34] | 2009 | China | No | NSCLC; BVI was not detected |
| [35] | 2009 | Japan | No | SCC, BVI was not detected |
| [36] | 2009 | France | No | Article in French |
| [37] | 2009 | Japan | No | Article in Japanese |
| [38] | 2009 | China | No | NSCLC; BVI was not detected |
| [39] | 2009 | Italy | Yes | NSCLC; survival analysis is not available |
| [40] | 2009 | USA | Yes | NSCLC; lymphatic or vascular invasion and survival |
| [41] | 2009 | Germany | No | NSCLC; BVI was not detected |
| [42] | 2008 | Japan | No | NSCLC; BVI was not detected |
| [43] | 2009 | UAE | No | Case report; Bronchioloalveolar carcinoma |
| [44] | 2008 | France | No | NSCLC; BVI was not detected |
| [45] | 2008 | Ireland | No | NSCLC; BVI was not detected |
| [46] | 2008 | China | Yes | Article in Chinese |
| [47] | 2008 | China | No | Live transplantation |
| [48] | 2008 | USA | No | Review |
| [49] | 2008 | Italy | Yes | NSCLC; survival analysis is not available |
| [50] | 2008 | Japan | No | NSCLC; BVI was not detected |
| [51] | 2008 | Turkey | Yes | NSCLC; survival analysis is not available |
| [52] | 2008 | Belgium | Yes | NSCLC; intra-tumoral permeation and survival |
| [53] | 2007 | Italy | Yes | NSCLC; blood vessel or lymphatic invasion and survival |
| [54] | 2007 | Spain | No | Review |
| [55] | 2007 | USA | No | Review |
| [56] | 2007 | Bosnia | No | NSCLC; BVI was not detected |
| [57] | 2007 | China | No | NSCLC; BVI was not detected |
| [58] | 2007 | China | No | NSCLC; BVI was not detected |
| [59] | 2007 | Japan | No | NSCLC; BVI was not detected |
| [60] | 2007 | Japan | Yes | NSCLC; survival analysis is not available |
| [61] | 2007 | China | No | NSCLC; BVI was not detected |
| [62] | 2007 | Japan | Yes | NSCLC; lymphatic or vascular invasion and survival |
| [63] | 2007 | China | No | NSCLC; BVI was not detected |
| [64] | 2006 | USA | No | Review |
| [65] | 2007 | Japan | Yes | NSCLC; lymphatic or vascular invasion and survival |
| [66] | 2007 | China | Yes | Article in Chinese |
| [67] | 2007 | Italy | No | NSCLC; BVI was not detected |
| [68] | 2007 | Japan | Yes | NSCLC, survival analysis is not available |
| [69] | 2007 | China | Yes | SCLC |
| [70] | 2006 | Italy | No | NSCLC; BVI was not detected |
| [71] | 2006 | USA | No | NSCLC; BVI was not detected |
| [72] | 2006 | USA | No | Review |
| [73] | 2006 | USA | No | NSCLC; BVI was not detected |
| [74] | 2006 | USA | No | NSCLC; BVI was not detected |
| [75] | 2006 | Japan | No | NSCLC; BVI was not detected |
| [76] | 2006 | Norway | No | Review |
| [77] | 2005 | Japan | No | Case report |
| [78] | 2005 | China | Yes | Article in Chinese |
| [79] | 2005 | France | Yes | NSCLC; lymphatic or vascular invasion and survival |
| [80] | 2005 | Japan | No | NSCLC; BVI was not detected |
| [81] | 2004 | Russia | Yes | Article in Russian |
| [82] | 2004 | Greece | No | Review |
| [83] | 2004 | Korea | No | NSCLC; BVI was not detected |
| [84] | 2004 | USA | No | NSCLC; BVI was not detected |
| [85] | 2004 | China | No | NSCLC; BVI was not detected |
| [86] | 2004 | USA | No | Review |
| [87] | 2004 | Germany | No | Review |
| [88] | 2004 | USA | No | Review |
| [89] | 2004 | Canada | No | NSCLC; BVI was not detected |
| [90] | 2004 | Greece | No | SCC; BVI was not detected |
| [91] | 2004 | Japan | No | NSCLC; BVI was not detected |
| [92] | 2004 | France | Yes | Articles in French |
| [93] | 2004 | Turkey | No | NSCLC; BVI was not detected |
| [94] | 2004 | Japan | No | NSCLC; BVI was not detected |
| [95] | 2004 | Japan | No | NSCLC; BVI was not detected |
| [96] | 2004 | USA | No | Review |
| [97] | 2004 | Japan | No | NSCLC; BVI was not detected |
| [98] | 2004 | China | Yes | Article in Chinese |
| [99] | 2004 | Turkey | Yes | NSCLC; survival analysis is not available |
| [100] | 2003 | Germany | No | Review |
| [101] | 2003 | USA | No | Review |
| [102] | 2003 | USA | No | Review |
| [103] | 2003 | Greece | No | NSCLC; BVI was not detected |
| [104] | 2003 | Japan | No | NSCLC; BVI was not detected |
| [105] | 2003 | China | No | NSCLC; BVI was not detected |
| [106] | 2003 | France | No | Review |
| [107] | 2003 | China | No | NSCLC; BVI was not detected |
| [108] | 2003 | Italy | No | BAC; BVI was not detected |
| [109] | 2003 | China | No | NSCLC; BVI was not detected |
| [110] | 2002 | China | No | NSCLC; BVI was not detected |
| [111] | 2002 | China | No | NSCLC; BVI was not detected |
| [112] | 2002 | France | No | NSCLC; BVI was not detected |
| [113] | 2001 | USA | No | Review |
| [114] | 2002 | Italy | No | NSCLC; BVI was not detected |
| [115] | 2002 | China | No | NSCLC; BVI was not detected |
| [116] | 2001 | China | No | NSCLC; BVI was not detected |
| [117] | 2001 | Japan | No | NSCLC; BVI was not detected |
| [107] | 2003 | China | No | NSCLC; BVI was not detected |
| [118] | 1999 | China | No | NSCLC; BVI was not detected |
| [119] | 2001 | Japan | No | Review |
| [120] | 2001 | USA | No | NSCLC; BVI was not detected |
| [121] | 2001 | China | No | NSCLC; BVI was not detected |
| [122] | 2001 | USA | Yes | Lymphovascular invasion and survival |
| [123] | 2000 | Czech | No | Article in Czech |
| [124] | 2000 | China | Yes | NSCLC; survival analysis is not available |
| [125] | 2000 | France | No | NSCLC; BVI was not detected |
| [126] | 2000 | Japan | No | AC; BVI was not detected |
| [127] | 2000 | USA | No | NSCLC; BVI was not detected |
| [128] | 1999 | Greece | No | NSCLC; BVI was not detected |
| [129] | 1999 | Japan | No | AC; BVI was not detected |
| [130] | 1998 | France | No | NSCLC; BVI was not detected |
| [131] | 1998 | Greece |  | NSCLC; BVI was not detected |
| [132] | 1998 | Japan | No | NSCLC; BVI was not detected |
| [133] | 1998 | Canada | No | NSCLC; BVI was not detected |
| [134] | 1997 | Japan | Yes | NSCLC; survival analysis is not available |
| [135] | 1997 | Japan | No | NSCLC; BVI was not detected |
| [136] | 1997 | Italy | No | NSCLC; BVI was not detected |
| [137] | 1997 | Japan | No | Article in Japanese |
| [138] | 1996 | Italy | No | NSCLC; BVI was not detected |
| [139] | 1996 | Japan | No | NSCLC; BVI was not detected |
| [140] | 1995 | Italy | No | NSCLC; BVI was not detected |
| [141] | 1995 | Japan | No | NSCLC; BVI was not detected |
| [142] | 1995 | France | No | NSCLC; BVI was not detected |
| [143] | 1994 | Japan | No | NSCLC; BVI was not detected |
| [144] | 1994 | France | No | NSCLC; BVI was not detected |
| [145] | 1994 | France | No | Article in French |
| [146] | 1993 | Japan | No | NSCLC; BVI was not detected |
| [147] | 1993 | Japan | Yes | NSCLC; survival analysis is not available |
| [148] | 1990 | Japan | No | Article in Japanese |
| [149] | 1980 | Japan | No | NSCLC; BVI was not detected |

NSCLC = non-small cell lung cancer; SCLC = small cell lung cancer; AC = adenocarcinoma; SCC = squamous cell cancer; BAC = Bronchioloalveolar lung cancer; BVI = blood vessel invasion; UAE, United Arab Emirates.

**References**

1. Funai K, Sugimura H, Morita T, Shundo Y, Shimizu K, et al. (2011) Lymphatic Vessel Invasion is a Significant Prognostic Indicator in Stage IA Lung Adenocarcinoma. Ann Surg Oncol.

2. Cedres S, Quispe I, Martinez P, Longo M, Rodriguez E, et al. (2010) Computed tomography (CT) predicts accurately the pathologic tumour size in stage I non-small-cell lung cancer (NSCLC). Clin Transl Oncol 12: 829-835.

3. Sakai Y, Ohbayashi C, Kanomata N, Kajimoto K, Sakuma T, et al. (2011) Significance of microscopic invasion into hilar peribronchovascular soft tissue in resection specimens of primary non-small cell lung cancer. Lung Cancer 73: 89-95.

4. Min KH, Park SJ, Lee KS, Hwang SH, Kim SR, et al. (2011) Clinical usefulness of D2-40 in non-small cell lung cancer. Lung 189: 57-63.

5. Marcus AI, Zhou W (2010) LKB1 regulated pathways in lung cancer invasion and metastasis. J Thorac Oncol 5: 1883-1886.

6. Igai H, Matsuura N, Tarumi S, Chang SS, Misaki N, et al. (2011) Clinicopathological study of p-T1aN0M0 non-small-cell lung cancer, as defined in the seventh edition of the TNM classification of malignant tumors. Eur J Cardiothorac Surg 39: 963-967.

7. Kitano H, Kageyama S, Hewitt SM, Hayashi R, Doki Y, et al. (2010) Podoplanin expression in cancerous stroma induces lymphangiogenesis and predicts lymphatic spread and patient survival. Arch Pathol Lab Med 134: 1520-1527.

8. Martinelli E, Troiani T, Morgillo F, Rodolico G, Vitagliano D, et al. (2010) Synergistic antitumor activity of sorafenib in combination with epidermal growth factor receptor inhibitors in colorectal and lung cancer cells. Clin Cancer Res 16: 4990-5001.

9. Li YM, Zhu SC, Liu ZK, Song CL, Wang YX, et al. (2010) [Characteristics of the lymph node metastases and influencing factors and their value in target region delineation in postoperative radiotherapy for thoracic esophageal carcinoma]. Zhonghua Zhong Liu Za Zhi 32: 391-395.

10. Yamaguchi Y, Ishii G, Kojima M, Yoh K, Otsuka H, et al. (2010) Histopathologic features of the tumor budding in adenocarcinoma of the lung: tumor budding as an index to predict the potential aggressiveness. J Thorac Oncol 5: 1361-1368.

11. Chambers A, Routledge T, Bille A, Scarci M (2010) Does surgery have a role in T4N0 and T4N1 lung cancer? Interact Cardiovasc Thorac Surg 11: 473-479.

12. Saito H, Kameda Y, Masui K, Murakami S, Kondo T, et al. (2011) Correlations between thin-section CT findings, histopathological and clinical findings of small pulmonary adenocarcinomas. Lung Cancer 71: 137-143.

13. Lin Q, Li M, Shen ZY, Xiong LW, Pan XF, et al. (2010) Prognostic impact of vascular endothelial growth factor-A and E-cadherin expression in completely resected pathologic stage I non-small cell lung cancer. Jpn J Clin Oncol 40: 670-676.

14. Kilicgun A, Turna A, Sayar A, Solak O, Urer N, et al. (2010) Very important histopathological factors in patients with resected non-small cell lung cancer: necrosis and perineural invasion. Thorac Cardiovasc Surg 58: 93-97.

15. Feng Y, Wang W, Hu J, Ma J, Zhang Y, et al. (2010) Expression of VEGF-C and VEGF-D as significant markers for assessment of lymphangiogenesis and lymph node metastasis in non-small cell lung cancer. Anat Rec (Hoboken) 293: 802-812.

16. Haura EB, Tanvetyanon T, Chiappori A, Williams C, Simon G, et al. (2010) Phase I/II study of the Src inhibitor dasatinib in combination with erlotinib in advanced non-small-cell lung cancer. J Clin Oncol 28: 1387-1394.

17. Sarkar S, Mazumdar A, Dash R, Sarkar D, Fisher PB, et al. (2010) ZD6474, a dual tyrosine kinase inhibitor of EGFR and VEGFR-2, inhibits MAPK/ERK and AKT/PI3-K and induces apoptosis in breast cancer cells. Cancer Biol Ther 9: 592-603.

18. Kadota K, Huang CL, Liu D, Nakashima N, Yokomise H, et al. (2010) The clinical significance of the tumor cell D2-40 immunoreactivity in non-small cell lung cancer. Lung Cancer 70: 88-93.

19. Ito S, Miyahara R, Takahashi R, Nagai S, Takenaka K, et al. (2009) Stromal aminopeptidase N expression: correlation with angiogenesis in non-small-cell lung cancer. Gen Thorac Cardiovasc Surg 57: 591-598.

20. Matsuyama M, Chijiwa T, Inoue Y, Abe Y, Nishi M, et al. (2009) Alternative splicing variant of vascular endothelial growth factor-A is a critical prognostic factor in non-small cell lung cancer. Oncol Rep 22: 1407-1413.

21. Yano T, Morodomi Y, Ito K, Yoshida T, Haro A, et al. (2010) Verification of the newly proposed T category (seventh edition of the tumor, node, and metastasis classification) from a clinicopathological viewpoint in non-small cell lung cancer-special reference to tumor size. J Thorac Oncol 5: 45-48.

22. Westeel V, Maitre J (2009) [Treatment of metastatic non small cell lung cancer]. Rev Prat 59: 949-951, 954.

23. Zhou Q, Suzuki K, Anami Y, Oh S, Takamochi K (2010) Clinicopathologic features in resected subcentimeter lung cancer--status of lymph node metastases. Interact Cardiovasc Thorac Surg 10: 53-57.

24. Sanli M, Isik AF, Tuncozgur B, Arslan E, Elbeyli L (2009) Resection via median sternotomy in patients with lung cancer invading the main pulmonary artery. Acta Chir Belg 109: 484-488.

25. Takenaka T, Yano T, Ito K, Morodomi Y, Miura N, et al. (2009) Biological significance of the maximum standardized uptake values on positron emission tomography in non-small cell lung cancer. J Surg Oncol 100: 688-692.

26. Ko YH, Jung CK, Lee MA, Byun JH, Kang JH, et al. (2008) Clinical significance of vascular endothelial growth factors (VEGF)-C and -D in resected non-small cell lung cancer. Cancer Res Treat 40: 133-140.

27. Yang HX, Hou X, Lin P, Rong TH, Yang H, et al. (2009) Survival and risk factors of surgically treated mediastinal invasion T4 non-small cell lung cancer. Ann Thorac Surg 88: 372-378.

28. Takeuchi K, Ogata S, Nakanishi K, Ozeki Y, Hiroi S, et al. (2010) LAT1 expression in non-small-cell lung carcinomas: analyses by semiquantitative reverse transcription-PCR (237 cases) and immunohistochemistry (295 cases). Lung Cancer 68: 58-65.

29. Anami Y, Iijima T, Suzuki K, Yokota J, Minami Y, et al. (2009) Bronchioloalveolar carcinoma (lepidic growth) component is a more useful prognostic factor than lymph node metastasis. J Thorac Oncol 4: 951-958.

30. Krupitskaya Y, Wakelee HA (2009) Ramucirumab, a fully human mAb to the transmembrane signaling tyrosine kinase VEGFR-2 for the potential treatment of cancer. Curr Opin Investig Drugs 10: 597-605.

31. Schuurbiers OC, Kaanders JH, van der Heijden HF, Dekhuijzen RP, Oyen WJ, et al. (2009) The PI3-K/AKT-pathway and radiation resistance mechanisms in non-small cell lung cancer. J Thorac Oncol 4: 761-767.

32. Li Z, Yu Y, Lu J, Luo Q, Wu C, et al. (2009) Analysis of the T descriptors and other prognosis factors in pathologic stage I non-small cell lung cancer in China. J Thorac Oncol 4: 702-709.

33. Tong X, Li K, Luo Z, Lu B, Liu X, et al. (2009) Decreased TIP30 expression promotes tumor metastasis in lung cancer. Am J Pathol 174: 1931-1939.

34. Chen CH, Lai JM, Chou TY, Chen CY, Su LJ, et al. (2009) VEGFA upregulates FLJ10540 and modulates migration and invasion of lung cancer via PI3K/AKT pathway. PLoS One 4: e5052.

35. Iwakiri S, Nagai S, Katakura H, Takenaka K, Date H, et al. (2009) D2-40-positive lymphatic vessel density is a poor prognostic factor in squamous cell carcinoma of the lung. Ann Surg Oncol 16: 1678-1685.

36. Perol M, Arpin D (2009) [Bevacizumab and non-small cell lung cancer: a new step?]. Rev Mal Respir 26: 125-138.

37. Sano I, Hara S, Matsumoto K, Hatachi G, Nakamura A, et al. (2009) [Clinical analysis of resected pulmonary pleomorphic carcinoma]. Kyobu Geka 62: 187-191.

38. Sun JG, Wang Y, Chen ZT, Zhuo WL, Zhu B, et al. (2009) Detection of lymphangiogenesis in non-small cell lung cancer and its prognostic value. J Exp Clin Cancer Res 28: 21.

39. Ruffini E, Asioli S, Filosso PL, Lyberis P, Bruna MC, et al. (2009) Clinical significance of tumor-infiltrating lymphocytes in lung neoplasms. Ann Thorac Surg 87: 365-371; discussion 371-362.

40. Varlotto JM, Recht A, Flickinger JC, Medford-Davis LN, Dyer AM, et al. (2009) Factors associated with local and distant recurrence and survival in patients with resected nonsmall cell lung cancer. Cancer 115: 1059-1069.

41. Wex P, Graeter T, Zaraca F, Haas V, Decker S, et al. (2009) Surgical resection and survival of patients with unsuspected single node positive lung cancer (NSCLC) invading the descending aorta. Thorac Surg Sci 6: Doc02.

42. Matsuda Y, Yamamoto T, Kudo M, Kawahara K, Kawamoto M, et al. (2008) Expression and roles of lumican in lung adenocarcinoma and squamous cell carcinoma. Int J Oncol 33: 1177-1185.

43. Al-Salam S, Al-Ashari M (2009) Expression of Galectin-3, CD138, p16INK4a, and TTF-1 in mucinous bronchioloalveolar adenocarcinoma after Hodgkin lymphoma. Appl Immunohistochem Mol Morphol 17: 351-356.

44. Yildizeli B, Dartevelle PG, Fadel E, Mussot S, Chapelier A (2008) Results of primary surgery with T4 non-small cell lung cancer during a 25-year period in a single center: the benefit is worth the risk. Ann Thorac Surg 86: 1065-1075; discussion 1074-1065.

45. Al-Sarraf N, Gately K, Lucey J, Aziz R, Doddakula K, et al. (2008) Clinical implication and prognostic significance of standardised uptake value of primary non-small cell lung cancer on positron emission tomography: analysis of 176 cases. Eur J Cardiothorac Surg 34: 892-897.

46. Gao YS, Xing XZ, Shao K, Feng XL, He J (2008) [Analysis of prognostic factors in 1826 patients with completely resected non-small cell lung cancer]. Zhonghua Zhong Liu Za Zhi 30: 134-137.

47. Man K, Lo CM, Xiao JW, Ng KT, Sun BS, et al. (2008) The significance of acute phase small-for-size graft injury on tumor growth and invasiveness after liver transplantation. Ann Surg 247: 1049-1057.

48. Gettinger S (2008) Targeted therapy in advanced non-small-cell lung cancer. Semin Respir Crit Care Med 29: 291-301.

49. Oliaro A, Filosso PL, Cavallo A, Giobbe R, Mossetti C, et al. (2008) The significance of intrapulmonary metastasis in non-small cell lung cancer: upstaging or downstaging? A re-appraisal for the next TNM staging system. Eur J Cardiothorac Surg 34: 438-443; discussion 443.

50. Kadota K, Huang CL, Liu D, Ueno M, Kushida Y, et al. (2008) The clinical significance of lymphangiogenesis and angiogenesis in non-small cell lung cancer patients. Eur J Cancer 44: 1057-1067.

51. Demir A, Gunluoglu MZ, Kara HV, Buyukpinarbasili N, Dincer SI (2008) Prognostic factors in resected T3 non-small cell lung carcinoma: perineural invasion as a new prognostic factor. Thorac Cardiovasc Surg 56: 93-98.

52. Poncelet AJ, Cornet J, Coulon C, Collard P, Noirhomme P, et al. (2008) Intra-tumoral vascular or perineural invasion as prognostic factors for long-term survival in early stage non-small cell lung carcinoma. Eur J Cardiothorac Surg 33: 799-804.

53. Rena O, Carsana L, Cristina S, Papalia E, Massera F, et al. (2007) Lymph node isolated tumor cells and micrometastases in pathological stage I non-small cell lung cancer: prognostic significance. Eur J Cardiothorac Surg 32: 863-867.

54. Quesada AR, Medina MA, Alba E (2007) Playing only one instrument may be not enough: limitations and future of the antiangiogenic treatment of cancer. Bioessays 29: 1159-1168.

55. Arenberg D (2007) Bronchioloalveolar lung cancer: ACCP evidence-based clinical practice guidelines (2nd edition). Chest 132: 306S-313S.

56. Radovic S, Babic M, Doric M, Hukic A, Kuskunovic S, et al. (2007) Non-small cell lung carcinoma: cyclin D1, bcl-2, p53, Ki-67 and HER-2 proteins expression in resected tumors. Bosn J Basic Med Sci 7: 205-211.

57. Chang YL, Wu CT, Lee YC (2007) Surgical treatment of synchronous multiple primary lung cancers: experience of 92 patients. J Thorac Cardiovasc Surg 134: 630-637.

58. Hong TM, Chen YL, Wu YY, Yuan A, Chao YC, et al. (2007) Targeting neuropilin 1 as an antitumor strategy in lung cancer. Clin Cancer Res 13: 4759-4768.

59. Mizuguchi S, Nishiyama N, Iwata T, Nishida T, Izumi N, et al. (2007) Serum Sialyl Lewis x and cytokeratin 19 fragment as predictive factors for recurrence in patients with stage I non-small cell lung cancer. Lung Cancer 58: 369-375.

60. Aokage K, Ishii G, Nagai K, Kawai O, Naito Y, et al. (2007) Intrapulmonary metastasis in resected pathologic stage IIIB non-small cell lung cancer: possible contribution of aerogenous metastasis to the favorable outcome. J Thorac Cardiovasc Surg 134: 386-391.

61. Guo CB, Wang S, Deng C, Zhang DL, Wang FL, et al. (2007) Relationship between matrix metalloproteinase 2 and lung cancer progression. Mol Diagn Ther 11: 183-192.

62. Tsuchiya T, Hashizume S, Akamine S, Muraoka M, Honda S, et al. (2007) Upstaging by vessel invasion improves the pathology staging system of non-small cell lung cancer. Chest 132: 170-177.

63. Lee YC, Wu CT, Kuo SW, Tseng YT, Chang YL (2007) Significance of extranodal extension of regional lymph nodes in surgically resected non-small cell lung cancer. Chest 131: 993-999.

64. Christiani DC, Pao W, DeMartini JC, Linnoila RI, Malkinson AM, et al. (2006) BAC consensus conference, November 4-6, 2004: epidemiology, pathogenesis, and preclinical models. J Thorac Oncol 1: S2-7.

65. Tsuchiya T, Akamine S, Muraoka M, Kamohara R, Tsuji K, et al. (2007) Stage IA non-small cell lung cancer: vessel invasion is a poor prognostic factor and a new target of adjuvant chemotherapy. Lung Cancer 56: 341-348.

66. Zhou S, Xu S, Zhang H, Liu Z, Liang Z, et al. (2007) [Prognostic significance of angiogenesis and blood vessel invasion in stage I non-small cell lung cancer after complete surgical resection.]. Zhongguo Fei Ai Za Zhi 10: 29-33.

67. Pompeo E, Tacconi F, Mineo TC (2007) Flexible videopericardioscopy in cT4 nonsmall-cell lung cancer with radiologic evidence of proximal vascular invasion. Ann Thorac Surg 83: 402-408.

68. Tsutsumida H, Goto M, Kitajima S, Kubota I, Hirotsu Y, et al. (2007) MUC4 expression correlates with poor prognosis in small-sized lung adenocarcinoma. Lung Cancer 55: 195-203.

69. Zhao J, Ni H, Ma Y, Dong L, Dai J, et al. (2007) TIP30/CC3 expression in breast carcinoma: relation to metastasis, clinicopathologic parameters, and P53 expression. Hum Pathol 38: 293-298.

70. Ghio P, Cappia S, Selvaggi G, Novello S, Lausi P, et al. (2006) Prognostic role of protease-activated receptors 1 and 4 in resected stage IB non-small-cell lung cancer. Clin Lung Cancer 7: 395-400.

71. D'Amico TA, Brooks KR, Joshi MB, Conlon D, Herndon J, 2nd, et al. (2006) Serum protein expression predicts recurrence in patients with early-stage lung cancer after resection. Ann Thorac Surg 81: 1982-1987; discussion 1987.

72. Raz DJ, He B, Rosell R, Jablons DM (2006) Bronchioloalveolar carcinoma: a review. Clin Lung Cancer 7: 313-322.

73. Stabile LP, Lyker JS, Land SR, Dacic S, Zamboni BA, et al. (2006) Transgenic mice overexpressing hepatocyte growth factor in the airways show increased susceptibility to lung cancer. Carcinogenesis 27: 1547-1555.

74. Zhang J, Ren H, Yuan P, Lang W, Zhang L, et al. (2006) Down-regulation of hepatoma-derived growth factor inhibits anchorage-independent growth and invasion of non-small cell lung cancer cells. Cancer Res 66: 18-23.

75. Takanami I (2006) Lymphatic microvessel density using D2-40 is associated with nodal metastasis in non-small cell lung cancer. Oncol Rep 15: 437-442.

76. Bremnes RM, Camps C, Sirera R (2006) Angiogenesis in non-small cell lung cancer: the prognostic impact of neoangiogenesis and the cytokines VEGF and bFGF in tumours and blood. Lung Cancer 51: 143-158.

77. Ohno K, Utsumi T, Sasaki Y, Suzuki Y (2005) Videopericardioscopy using endothoracic sonography for lung cancer staging. Ann Thorac Surg 79: 1780-1782.

78. Yu M, Li SY, Yu Z, Qiu XS, Hou P, et al. (2005) [Clinical significance of heparanase and basic fibroblast growth factor expression in human non-small cell lung cancer]. Zhonghua Bing Li Xue Za Zhi 34: 36-41.

79. Doddoli C, Aragon A, Barlesi F, Chetaille B, Robitail S, et al. (2005) Does the extent of lymph node dissection influence outcome in patients with stage I non-small-cell lung cancer? Eur J Cardiothorac Surg 27: 680-685.

80. Higashi K, Ito K, Hiramatsu Y, Ishikawa T, Sakuma T, et al. (2005) 18F-FDG uptake by primary tumor as a predictor of intratumoral lymphatic vessel invasion and lymph node involvement in non-small cell lung cancer: analysis of a multicenter study. J Nucl Med 46: 267-273.

81. Akopov AL, Dvorakovskaia IV (2004) [Vascular invasion by tumor in the absence of regional lymph node metastases in patients with locally advanced non-small cell lung cancer]. Vopr Onkol 50: 417-420.

82. Giatromanolaki A, Sivridis E, Koukourakis MI (2004) Tumour angiogenesis: vascular growth and survival. APMIS 112: 431-440.

83. Lee KS, Jeong YJ, Han J, Kim BT, Kim H, et al. (2004) T1 non-small cell lung cancer: imaging and histopathologic findings and their prognostic implications. Radiographics 24: 1617-1636; discussion 1632-1616.

84. Pechet TT, Carr SR, Collins JE, Cohn HE, Farber JL (2004) Arterial invasion predicts early mortality in stage I non-small cell lung cancer. Ann Thorac Surg 78: 1748-1753.

85. Zhang L, Meng L, Wang L, Peng Z, Chen J, et al. (2004) [The clinical significance of detection of vascular endothelial growth factor and CD44v6 expression in human non-small cell lung cancer.]. Zhongguo Fei Ai Za Zhi 7: 427-430.

86. Sabloff BS, Truong MT, Wistuba, II, Erasmus JJ (2004) Bronchioalveolar cell carcinoma: radiologic appearance and dilemmas in the assessment of response. Clin Lung Cancer 6: 108-112.

87. Macchiarini P (2004) Resection of superior sulcus carcinomas (anterior approach). Thorac Surg Clin 14: 229-240.

88. Raben D, Helfrich B (2004) Angiogenesis inhibitors: a rational strategy for radiosensitization in the treatment of non-small-cell lung cancer? Clin Lung Cancer 6: 48-57.

89. Shargall Y, de Perrot M, Keshavjee S, Darling G, Ginsberg R, et al. (2004) 15 years single center experience with surgical resection of the superior vena cava for non-small cell lung cancer. Lung Cancer 45: 357-363.

90. Pentheroudakis G, Kostadima L, Fountzilas G, Kalogera-Fountzila A, Klouvas G, et al. (2004) Cavitating squamous cell lung carcinoma-distinct entity or not? Analysis of radiologic, histologic, and clinical features. Lung Cancer 45: 349-355.

91. Moriya Y, Iyoda A, Hiroshima K, Sekine Y, Shibuya K, et al. (2004) Clinicopathological analysis of clinical N0 peripheral lung cancers with a diameter of 1 cm or less. Thorac Cardiovasc Surg 52: 196-199.

92. Barlesi F, Doddoli C, Greillier L, Astoul P, Giudicelli R, et al. (2004) [Prognostic indicators in stage I non-small cell lung cancer]. Rev Mal Respir 21: 93-103.

93. Kaya A, Ciledag A, Gulbay BE, Poyraz BM, Celik G, et al. (2004) The prognostic significance of vascular endothelial growth factor levels in sera of non-small cell lung cancer patients. Respir Med 98: 632-636.

94. Yamamoto S, Tomita Y, Hoshida Y, Iizuka N, Monden M, et al. (2004) Expression level of valosin-containing protein (p97) is correlated with progression and prognosis of non-small-cell lung carcinoma. Ann Surg Oncol 11: 697-704.

95. Takenaka K, Ishikawa S, Kawano Y, Yanagihara K, Miyahara R, et al. (2004) Expression of a novel matrix metalloproteinase regulator, RECK, and its clinical significance in resected non-small cell lung cancer. Eur J Cancer 40: 1617-1623.

96. Raben D, Helfrich B, Bunn PA, Jr. (2004) Targeted therapies for non-small-cell lung cancer: biology, rationale, and preclinical results from a radiation oncology perspective. Int J Radiat Oncol Biol Phys 59: 27-38.

97. Iwasaki A, Kuwahara M, Yoshinaga Y, Shirakusa T (2004) Basic fibroblast growth factor (bFGF) and vascular endothelial growth factor (VEGF) levels, as prognostic indicators in NSCLC. Eur J Cardiothorac Surg 25: 443-448.

98. Yu M, Dong X, Li S, Hou P, Long F, et al. (2004) [Expression of heparanase in human non-small cell lung cancer.]. Zhongguo Fei Ai Za Zhi 7: 16-21.

99. Sayar A, Turna A, Solak O, Kilicgun A, Urer N, et al. (2004) Nonanatomic prognostic factors in resected nonsmall cell lung carcinoma: the importance of perineural invasion as a new prognostic marker. Ann Thorac Surg 77: 421-425.

100. Abdollahi A, Lipson KE, Sckell A, Zieher H, Klenke F, et al. (2003) Combined therapy with direct and indirect angiogenesis inhibition results in enhanced antiangiogenic and antitumor effects. Cancer Res 63: 8890-8898.

101. Sandler AB (2003) Molecular targeted agents in non-small-cell lung cancer. Clin Lung Cancer 5 Suppl 1: S22-28.

102. Choy H, Milas L (2003) Enhancing radiotherapy with cyclooxygenase-2 enzyme inhibitors: a rational advance? J Natl Cancer Inst 95: 1440-1452.

103. Koukourakis MI, Giatromanolaki A, Brekken RA, Sivridis E, Gatter KC, et al. (2003) Enhanced expression of SPARC/osteonectin in the tumor-associated stroma of non-small cell lung cancer is correlated with markers of hypoxia/acidity and with poor prognosis of patients. Cancer Res 63: 5376-5380.

104. Sato J, Sata M, Nakamura H, Inoue S, Wada T, et al. (2003) Role of thymidine phosphorylase on invasiveness and metastasis in lung adenocarcinoma. Int J Cancer 106: 863-870.

105. Li Q, Dong X, Gu W, Qiu X, Wang E (2003) Clinical significance of co-expression of VEGF-C and VEGFR-3 in non-small cell lung cancer. Chin Med J (Engl) 116: 727-730.

106. Barlesi F, Doddoli C, Gimenez C, Chetaille B, Giudicelli R, et al. (2003) Bronchioloalveolar carcinoma: myths and realities in the surgical management. Eur J Cardiothorac Surg 24: 159-164.

107. Dong X, Qiu XS, Wang EH, Li QC, Gu W (2003) [Expression of vascular endothelial growth factor (VEGF) C and VEGF receptor 3 in non-small cell lung cancer]. Zhonghua Bing Li Xue Za Zhi 32: 128-132.

108. Rena O, Papalia E, Ruffini E, Casadio C, Filosso PL, et al. (2003) Stage I pure bronchioloalveolar carcinoma: recurrences, survival and comparison with adenocarcinoma of the lung. Eur J Cardiothorac Surg 23: 409-414.

109. Liang RY, Liao ZS, Jiang SP, Zhang W, Li JG, et al. (2003) [Expression of cyclin D1 and vascular endothelial growth factor(VEGF) in non-small cell lung carcinoma and their association with the prognosis]. Ai Zheng 22: 86-90.

110. Wang Y, Zhang X, Tan W, Fu J, Zhang W (2002) [Significance of fatty acid synthase expression in non-small cell lung cancer]. Zhonghua Zhong Liu Za Zhi 24: 271-273.

111. Lin CM, Li AF, Wu LH, Wu YC, Lin FC, et al. (2002) Adenoid cystic carcinoma of the trachea and bronchus--a clinicopathologic study with DNA flow cytometric analysis and oncogene expression. Eur J Cardiothorac Surg 22: 621-625.

112. Fadel E, Yildizeli B, Chapelier AR, Dicenta I, Mussot S, et al. (2002) Sleeve lobectomy for bronchogenic cancers: factors affecting survival. Ann Thorac Surg 74: 851-858; discussion 858-859.

113. Wright CD, Mathisen DJ (2001) Superior sulcus tumors. Curr Treat Options Oncol 2: 43-49.

114. Spaggiari L, Thomas P, Magdeleinat P, Kondo H, Rollet G, et al. (2002) Superior vena cava resection with prosthetic replacement for non-small cell lung cancer: long-term results of a multicentric study. Eur J Cardiothorac Surg 21: 1080-1086.

115. Lee YC, Wu CT, Chen CS, Hsu HH, Chang YL (2002) The significance of E-cadherin and alpha-, beta-, and gamma-catenin expression in surgically treated non-small cell lung cancers of 3 cm or less in size. J Thorac Cardiovasc Surg 123: 502-507.

116. Fu X, Jiang G, Wu K, Zhu X, Shi D, et al. (2001) [Multivariate analysis for prognostic predictors in non-small cell lung cancer.]. Zhongguo Fei Ai Za Zhi 4: 459-462.

117. Shou Y, Hirano T, Gong Y, Kato Y, Yoshida K, et al. (2001) Influence of angiogenetic factors and matrix metalloproteinases upon tumour progression in non-small-cell lung cancer. Br J Cancer 85: 1706-1712.

118. Yang G, Zhang L, Xie Y (1999) [Vascular endothelial growth factor in non-small cell lung carcinoma expression and its significance]. Zhonghua Yi Xue Za Zhi 79: 104-105.

119. Iizasa T, Fujisawa T (2001) [Surgery for non-small cell lung cancer based on the T factor]. Nippon Geka Gakkai Zasshi 102: 507-510.

120. Khuri FR, Wu H, Lee JJ, Kemp BL, Lotan R, et al. (2001) Cyclooxygenase-2 overexpression is a marker of poor prognosis in stage I non-small cell lung cancer. Clin Cancer Res 7: 861-867.

121. Shen C, Fan S, Jiang Y, Chen J, Li Z (2001) [Effects of vascular endothelial growth factor in the invasion and metastasis of non-small cell lung cancer.]. Zhongguo Fei Ai Za Zhi 4: 91-93.

122. Han H, Silverman JF, Santucci TS, Macherey RS, d'Amato TA, et al. (2001) Vascular endothelial growth factor expression in stage I non-small cell lung cancer correlates with neoangiogenesis and a poor prognosis. Ann Surg Oncol 8: 72-79.

123. Klein J, Bohanes T, Tichy T, Kral V, Kolek V, et al. (2000) [Assessment of metastatic potential of pulmonary carcinoma]. Rozhl Chir 79: 460-463.

124. Lee YC, Wu CT, Chen CS, Chang YL (2000) E-cadherin expression in surgically-resected non-small cell lung cancers--a clinicopathological study. Thorac Cardiovasc Surg 48: 294-299.

125. Chapelier A, Fadel E, Macchiarini P, Lenot B, Le Roy Ladurie F, et al. (2000) Factors affecting long-term survival after en-bloc resection of lung cancer invading the chest wall. Eur J Cardiothorac Surg 18: 513-518.

126. Higashi K, Ueda Y, Ayabe K, Sakurai A, Seki H, et al. (2000) FDG PET in the evaluation of the aggressiveness of pulmonary adenocarcinoma: correlation with histopathological features. Nucl Med Commun 21: 707-714.

127. Herbst RS, Yano S, Kuniyasu H, Khuri FR, Bucana CD, et al. (2000) Differential expression of E-cadherin and type IV collagenase genes predicts outcome in patients with stage I non-small cell lung carcinoma. Clin Cancer Res 6: 790-797.

128. Kakolyris S, Giatromanolaki A, Koukourakis M, Leigh IM, Georgoulias V, et al. (1999) Assessment of vascular maturation in non-small cell lung cancer using a novel basement membrane component, LH39: correlation with p53 and angiogenic factor expression. Cancer Res 59: 5602-5607.

129. Kawabuchi B, Moriyama S, Hironaka M, Fujii T, Koike M, et al. (1999) p16 inactivation in small-sized lung adenocarcinoma: its association with poor prognosis. Int J Cancer 84: 49-53.

130. Riquet M, Le Pimpec-Barthes F, Danel C (1998) Axillary lymph node metastases from bronchogenic carcinoma. Ann Thorac Surg 66: 920-922.

131. Koukourakis MI, Giatromanolaki A, Kakolyris S, O'Byrne KJ, Apostolikas N, et al. (1998) Different patterns of stromal and cancer cell thymidine phosphorylase reactivity in non-small-cell lung cancer: impact on tumour neoangiogenesis and survival. Br J Cancer 77: 1696-1703.

132. Hirata T, Fukuse T, Naiki H, Hitomi S, Wada H (1998) Expression of CD44 variant exon 6 in stage I non-small cell lung carcinoma as a prognostic factor. Cancer Res 58: 1108-1110.

133. Lacasse Y, Bucher HC, Wong E, Griffith L, Walter S, et al. (1998) "Incomplete resection" in non-small cell lung cancer: need for a new definition. Canadian Lung Oncology Group. Ann Thorac Surg 65: 220-226.

134. Guang SG, Ogura T, Sekine I, Yokozaki M, Esumi H, et al. (1997) Association between p53 mutation and clinicopathological features of non-small cell lung cancer. Jpn J Clin Oncol 27: 211-215.

135. Fukuse T, Wada H, Hitomi S (1997) Extended operation for non-small cell lung cancer invading great vessels and left atrium. Eur J Cardiothorac Surg 11: 664-669.

136. Fontanini G, Vignati S, Chine S, Lucchi M, Mussi A, et al. (1997) 67-Kilodalton laminin receptor expression correlates with worse prognostic indicators in non-small cell lung carcinomas. Clin Cancer Res 3: 227-231.

137. Sakurada A, Sagawa M, Usuda K, Kanda A, Aikawa H, et al. (1997) [The significance of surgical treatment for T4 lung cancer]. Kyobu Geka 50: 95-100.

138. Angeletti CA, Lucchi M, Fontanini G, Mussi A, Chella A, et al. (1996) Prognostic significance of tumoral angiogenesis in completely resected late stage lung carcinoma (stage IIIA-N2). Impact of adjuvant therapies in a subset of patients at high risk of recurrence. Cancer 78: 409-415.

139. Nakajima J, Furuse A, Oka T, Kohno T, Ohtsuka T (1996) Excellent survival in a subgroup of patients with intrapulmonary metastasis of lung cancer. Ann Thorac Surg 61: 158-162; discussion 162-153.

140. Fontanini G, Bigini D, Vignati S, Basolo F, Mussi A, et al. (1995) Microvessel count predicts metastatic disease and survival in non-small cell lung cancer. J Pathol 177: 57-63.

141. Ogawa J, Sano A, Inoue H, Koide S (1995) Expression of Lewis-related antigen and prognosis in stage I non-small cell lung cancer. Ann Thorac Surg 59: 412-415.

142. Riquet M, Manac'h D, Saab M, Le Pimpec-Barthes F, Dujon A, et al. (1995) Factors determining survival in resected N2 lung cancer. Eur J Cardiothorac Surg 9: 300-304.

143. Ogawa J, Sano A, Koide S, Shohtsu A (1994) Relation between recurrence and expression of proliferating cell nuclear antigen, sialyl LewisX, and sialyl Lewis(a) in lung cancer. J Thorac Cardiovasc Surg 108: 329-336.

144. Macchiarini P, Fontanini G, Dulmet E, de Montpreville V, Chapelier AR, et al. (1994) Angiogenesis: an indicator of metastasis in non-small cell lung cancer invading the thoracic inlet. Ann Thorac Surg 57: 1534-1539.

145. Riquet M, Manac'h D, Debrosse D, Lepimpec Barthes F, Saab M, et al. (1994) [Prognostic factors of survival in resected N2 bronchial cancer]. Ann Chir 48: 259-265.

146. Ichinose Y, Hara N, Ohta M, Yano T, Maeda K, et al. (1993) Is T factor of the TNM staging system a predominant prognostic factor in pathologic stage I non-small-cell lung cancer ? A multivariate prognostic factor analysis of 151 patients. J Thorac Cardiovasc Surg 106: 90-94.

147. Fujii M, Motoi M, Saeki H, Aoe K, Moriwaki S (1993) Prognostic significance of proliferating cell nuclear antigen (PCNA) expression in non-small cell lung cancer. Acta Med Okayama 47: 103-108.

148. Kodama T, Nishiyama H, Nishiwaki Y (1990) [Histology and prognosis in lung cancer treatment]. Nihon Kyobu Shikkan Gakkai Zasshi 28: 216-224.

149. Shimosato Y, Suzuki A, Hashimoto T, Nishiwaki Y, Kodama T, et al. (1980) Prognostic implications of fibrotic focus (scar) in small peripheral lung cancers. Am J Surg Pathol 4: 365-373.
