# Supplementary material for: Blood Vessel Invasion as a Strong Independent Prognostic Indicator in Non-Small Cell Lung Cancer: A Systematic Review and Meta-Analysis
Source: PLoS One. 2011 Dec 14;6(12):e28844. doi: 10.1371/journal.pone.0028844 (PMC3237541; doi:10.1371/journal.pone.0028844)
Supplement: Table S3 — Main characteristics and results of eligible studies evaluating blood vessel invasion and RFS or OS in patients with NSCLC. (DOC) [file pone.0028844.s005.doc]

**Table S3.** Main characteristics and results of eligible studies evaluating blood vessel invasion and RFS or OS in patients with NSCLC

| First author | Year | Country | Method for BVI evaluation | BVI (+) | N | Stage | Histology | RFS |  | OS |  | Evaluable |
| --- | --- | --- | --- | --- | --- | --- | --- | --- | --- | --- | --- | --- |
|  |  |  |  |  |  |  |  | Univariate | Multivariate analysis | Univariate | Multivariate analysis |  |
| Maeda *et al*. (60) | 2011 | Japan | H&E, VVG | 1144 | 2295 | I-III | NSCLC | S | S | N/A | N/A | Yes |
| Sakai *et al*. (56) | 2011 | Japan | H&E , EVG | 271 | 543 | I-IV | NSCLC | N/A | N/A | N/A | NS | Yes |
| Shao *et al*. (20) | 2011 | China | H&E | 71 | 146 | IA | NSCLC | N/A | N/A | S | S | Yes |
| Maeda *et al*. (10) | 2010 | Japan | H&E , VVG | 87 | 813 | I-III | NSCLC | S^a^ | S^a^ | N/A | S | Yes |
| Shimada *et al*. (21) | 2010 | Japan | H&E, VVG | 460 | 1000 | I-IV | NSCLC | S | N/A | S | NS | Yes |
| Maeda *et al*. (11) | 2010 | Japan | H&E, VVG, D2-40 | 19 | 96 | IA | NSCLC | S^a^ | S^a^ | N/A | N/A | Yes |
| Naito *et al*. (22) | 2010 | Japan | H&E, VVG | 317 | 885 | I-III | NSCLC | N/A | N/A | S | N/A | Yes |
| Yamaguchi *et al*. (23) | 2010 | Japan | H&E, VVG | 58 | 181 | I-IIIA | NSCLC | N/A | N/A | S | S | Yes |
| Maeda *et al*. (9) | 2010 | Japan | H&E, VVG | 241 | 434 | IB | NSCLC | N/A | N/A | S | S | Yes |
| Ryuge *et al*. (24) | 2010 | Japan | H&E | 80 | 160 | I-III | NSCLC | N/A | N/A | S | N/A | Yes |
| Bodendorf *et al*. (25) | 2009 | German | H&E | 51 | 112 | IIB-IIIB | NSCLC | N/A | N/A | S | N/A | Yes |
| Kawachi *et al*. (26) | 2009 | Japan | H&E | 112 | 568 | I-IV | NSCLC | N/A | N/A | N/A | S | Yes |
| Turhan *et al*. (12) | 2009 | Japan | H&E, CD31, CD34 | 13 | 71 | I | NSCLC | S | N/A | S | N/A | Yes |
| Shoji *et al*. (13) | 2009 | Japan | H&E , EVG | 19 | 217 | IA | NSCLC | S | S | N/A | N/A | Yes |
| Mizuno *et al*. (27) | 2008 | Japan | H&E, VVG | 46 | 106 | IB | AC | N/A | N/A | S | S | Yes |
| Kashiwabara *et al*. (14) | 2008 | Japan | H&E | 26 | 136 | I | AC | S | S | N/A | N/A | Yes |
| Hashizume *et al*. (28) | 2008 | Japan | H&E , EVG, D2-40 | 16 | 221 | I | NSCLC | N/A | N/A | S | NS | Yes |
| Kawachi *et al*. (15) | 2008 | Japan | H&E | 45 | 226 | I | NSCLC | N/A | S | N/A | N/A | Yes |
| Gao *et al*. (59) | 2008 | China | H&E | 33 | 259 | I-II | NSCLC | N/A | N/A | S | S | Yes |
| Rao *et al*. (29) | 2007 | Canada | H&E | 9 | 35 | I-IIIB | NSCLC | N/A | N/A | S | N/A | Yes |
| Matsuguma *et al*. (48) | 2007 | Japan | H&E , EVG | 145 | 455 | I | NSCLC | N/A | N/A | N/A | NS | Yes |
| Ayed *et al*. (3089) | 2006 | Kuwai | H&E | 33 | 98 | I-II | NSCLC | N/A | N/A | S | NS | No |
| Takanami *et al*. (31) | 2005 | Japan | H&E | 41 | 134 | I-IIIA | NSCLC | N/A | N/A | S | S | Yes |
| Shimizu *et al*. (58) | 2005 | Japan | H&E, VVG | 490 | 1074 | I-II | NSCLC | N/A | N/A | S | S | Yes |
| Barlesi *et al*. (32) | 2005 | France | H&E | 38 | 155 | III | NSCLC | N/A | N/A | S | S | No |
| Wu *et al*. (33) | 2005 | China | H&E | 70 | 301 | I-III | NSCLC | N/A | N/A | S | S | Yes |
| Yoshida *et al*. (49) | 2004 | Japan | H&E | 29 | 79 | I-III | NSCLC | N/A | N/A | N/A | NS | Yes |
| Mineo *et al*. (34) | 2004 | Italy | H&E, CD34 | 13 | 51 | IB-IIA | NSCLC | N/A | N/A | S | S | Yes |
| Yamamoto *et al*. (35) | 2004 | Japan | H&E | 16 | 204 | I-IIIA | NSCLC | NS | N/A | S | S | Yes |
| Okada *et al*. (16) | 2003 | Japan | H&E | 68 | 265 | I-IIIB | NSCLC | S | N/A | S | S^b^ | Yes |
| Okada *et al*. (36) | 2003 | Japan | H&E, EVG | 429 | 1000 | I-III | NSCLC | N/A | N/A | N/A | S | Yes |
| Gabor *et al*. (17) | 2003 | Austria | H&E | 11 | 72 | I-III | NSCLC | S | N/A | S | N/A | Yes |
| Khan *et al*. (37) | 2003 | UK | H&E | 10 | 98 | II | NSCLC | N/A | N/A | S | S | Yes |
| Rigau *et al*. (18) | 2002 | France | H&E | 25 | 86 | I-IV | NSCLC | S | NS | NS | NS | Yes |
| Moriya *et al*. (38) | 2001 | Japan | H&E | 43 | 102 | I-IV | AC | N/A | N/A | S | S | Yes |
| Tamura *et al*. (39) | 2001 | Japan | H&E, EVG | 19 | 36 | I | SCC | N/A | N/A | S | S | Yes |
| Thomas *et al*. (40) | 2001 | France | H&E | 32 | 515 | I | NSCLC | N/A | N/A | S | S | Yes |
| Yokose *et al*. (41) | 2000 | Japan | H&E, EVG | 72 | 200 | I | AC | N/A | N/A | S | S | Yes |
| Cagini *et al*. (50) | 2000 | Italy | H&E, CD34 | 22 | 99 | I-II | NSCLC | N/A | N/A | NS | N/A | Yes |
| Suzuki *et al*. (42) | 1999 | Japan | H&E | 461 | 832 | I | NSCLC | N/A | N/A | S | S | Yes |
| Fu *et a*l. (51) | 1999 | China | H&E | 121 | 158 | I-III | NSCLC | N/A | N/A | NS | NS | Yes |
| Hirata *et al* (52) | 1998 | Japan | H&E | 20 | 69 | I | NSCLC | N/A | N/A | NS | NS | Yes |
| Lucchi *et al* (53) | 1997 | Italy | H&E, CD34 | 36 | 227 | I | NSCLC | NS | N/A | NS | NS | No |
| Kessler *et al*. (43) | 1996 | France | H&E | 284 | 593 | I-III | NSCLC | N/A | N/A | S | S | Yes |
| Brechot *et al*. (54) | 1996 | France | H&E | 50 | 96 | I-IV | NSCLC | NS | NS | NS | N/A | Yes |
| Duarte *et al*. (44) | 1997 | USA | H&E | 11 | 105 | I | NSCLC | N/A | N/A | S | S | Yes |
| Fujisaw *et al*. (45) | 1995 | Japan | H&E, elastica staining | 29 | 66 | I-III | NSCLC | N/A | N/A | S | S | Yes |
| Harpole *et al*. (65) | 1995 | USA | H&E | 25 | 289 | I | NSCLC | N/A | N/A | S | S | Yes |
| Ichinose *et al*. (47) | 1994 | Japan | H&E, elastica staining | 27 | 243 | I-IIIA | NSCLC | N/A | N/A | S | S | Yes |
| Ogawa *et al*. *et al*. (19) | 1993 | Japan | H&E, elastica staining | 55 | 128 | I | NSCLC | S | S | N/A | N/A | Yes |
| Roberts *et al*. (55) | 1992 | UK | H&E, EVG | 67 | 87 | I-III | NSCLC | N/A | N/A | NS | N/A | No |
| Macchiarini *et al*. (8) | 1993 | Italy | H&E | 16 | 95 | I | NSCLC | S | S | S | S | Yes |

NSCLC = non-small cell lung cancer; AC = adenocarcinoma; N = overall cases; BVI (+) = cases for blood vessel invasion; S = significant relationship between BVI and relapse or survival; NS = no significant relationship between BVI and relapse or survival; N/A = no available or no applicable; RFS = relapse-free survival; OS = overall survival. H&E = hematoxylin and eosin stain; EVG = elastica van Gieson staining; VVG = Victoria blue-van Gieson staining. ^a^ Identical patient cohort occurred within another selected cohort (reference 161). ^b^ Identical patient cohort occurred within another selected cohort (reference 39).
